# Application of machine learning models to identify serological predictors of COVID-19 severity and outcomes

**DOI:** 10.21203/rs.3.rs-3463155/v1

**Published:** 2023-11-13

**Authors:** Sabra Klein, Santosh Dhakal, Anna Yin, Marta Escarra-Senmarti, Zoe Demko, Nora Pisanic, Trevor Johnston, Maria Trejo-Zambrano, Kate Kruczynski, John Lee, Justin Hardick, Patrick Shea, Janna Shapiro, Han-Sol Park, Maclaine Parish, Christopher Caputo, Abhinaya Ganesan, Sarika Mullapudi, Stephen Gould, Michael Betenbaugh, Andrew Pekosz, Christopher D Heaney, Annukka Antar, Yukari Manabe, Andrea Cox, Andrew Karaba, Felipe Andrade, Scott Zeger

**Affiliations:** Johns Hopkins Bloomberg School of Public Health; Johns Hopkins Bloomberg School of Public Health; Johns Hopkins Bloomberg School of Public Health; Johns Hopkins School of Medicine; Johns Hopkins School of Medicine; Johns Hopkins Bloomberg School of Public Health; Johns Hopkins Bloomberg School of Public Health; Johns Hopkins School of Medicine; Johns Hopkins Bloomberg School of Public Health; Johns Hopkins Bloomberg School of Public Health; The Johns Hopkins University; Johns Hopkins Bloomberg School of Public Health; Johns Hopkins Bloomberg School of Public Health; Johns Hopkins Bloomberg School of Public Health; Johns Hopkins Bloomberg School of Public Health; Johns Hopkins Bloomberg School of Public Health, Baltimore; Johns Hopkins Bloomberg School of Public Health; Johns Hopkins School of Medicine; Johns Hopkins University School of Medicine; Johns Hopkins University; Johns Hopkins Bloomberg School of Public Health; Johns Hopkins; Johns Hopkins School of Medicine; Division of Infectious Diseases, Department of Medicine, The Johns Hopkins School of Medicine; Johns Hopkins University; Johns Hopkins University; Johns Hopkins University; Johns Hopkins University

**Keywords:** automated intelligence, IgG isotypes, neutralizing antibody, non-neutralizing antibody, COVID-19 death, COVID-19 hospitalization, random forest model

## Abstract

Critically ill people with COVID-19 have greater antibody titers than those with mild to moderate illness, but their association with recovery or death from COVID-19 has not been characterized. In 178 COVID-19 patients, 73 non-hospitalized and 105 hospitalized patients, mucosal swabs and plasma samples were collected at hospital enrollment and up to 3 months post-enrollment (MPE) to measure virus RNA, cytokines/chemokines, binding antibodies, ACE2 binding inhibition, and Fc effector antibody responses against SARS-CoV-2. The association of demographic variables and >20 serological antibody measures with intubation or death due to COVID-19 was determined using machine learning algorithms. Predictive models revealed that IgG binding and ACE2 binding inhibition responses at 1 MPE were positively and C1q complement activity at enrollment was negatively associated with an increased probability of intubation or death from COVID-19 within 3 MPE. Serological antibody measures were more predictive than demographic variables of intubation or death among COVID-19 patients.

## Introduction

Most SARS-CoV-2 infections cause mild to moderate disease and do not require hospitalization^1^. Severe disease (i.e., hospitalization or intensive care unit (ICU) admission) and fatal outcomes are associated with older age, male sex, underlying comorbidities, and lack of vaccination^2,3^. Antibodies protect against SARS-CoV-2 and the development of neutralizing antibodies is the leading candidate for a correlate of protection. Non-neutralizing antibody responses mediated by the crystallizable fragment (Fc) region also are critical in COVID-19 pathogenesis^4,5^.

Epidemiological and vaccine studies have shown that anti-Spike (S) IgG, anti-S-receptor-binding domain (S-RBD) IgG, and neutralizing antibodies correlate with protection against SARS-CoV-2^6,7^. The role of antibodies in the control of SARS-CoV-2 infection and the pathogenesis of disease is still ambiguous as studies have consistently shown that both binding and neutralizing antibody titers are greater in patients with more severe COVID-19^8,9^. The greater magnitude of antibody titers is observed in severe COVID-19 patients both during the acute phase of the disease and convalescence^8,10^. The association of hospitalization and subsequent deaths in individuals with greater antibody responses raises questions about the role of antibodies in the protection versus pathogenesis of COVID-19. One study highlighted that the antibody repertoire in mild COVID-19 patients exhibits greater diversity, antibody class switching, and affinity maturation than in severe COVID-19 patients^11^. Despite having higher antibody titers, individuals with severe COVID-19 produce less potent and functional antibodies, thereby contributing to pathogenesis^12^.

Despite known variations in the quantity and quality of antibody responses based on disease severity, the antibody dynamics that predict COVID-19 progression (i.e., survival or recovery) are still unclear. Most studies typically measure antibody responses in serum or plasma, but mucosal immunity to SARS-CoV-2, either in respiratory or oral fluid samples, may provide a better correlate of protection. Using a longitudinal cohort at Johns Hopkins Hospital, we analyzed antibody responses in plasma and mucosal samples, measured proinflammatory cytokines and chemokines in plasma, and determined the associations of key demographic variables and antibody responses with COVID-19 outcome.

## Methods

### Study cohorts

A convenience sample of hospitalized (n=105) and non-hospitalized (n=73) patients were enrolled into a prospective cohort study from April 2020 through April 2021 (**Table 1**). This study was approved by the Institutional Review Board (IRB) of the Johns Hopkins University (IRB00245545, IRB00259948)^13–15^. Verbal consent was obtained using consent waiver with an alteration of informed consent from all non-hospitalized participants, and written consent was obtained from all hospitalized participants. The study comprised Johns Hopkins Hospital in- or out-patients who were 18 years or older with reference lab RT-PCR-confirmed SARS-CoV-2 diagnosis. Blood plasma samples were collected from non-hospitalized patients at one-month post-enrollment (MPE). The 1 MPE for non-hospitalized patients ranged between 18 to 91 days after PCR-confirmation, averaging at 46 ± 15 days, and antibody levels were comparable among non-hospitalized patients within this time frame (**Supplementary Figure 1A-B**). Blood plasma samples were collected from hospitalized patients at study enrollment, 1 MPE, and until subsequent death or up until 100 days post-enrollment (DPE) (**Supplementary Figure 1C-D**). Samples from hospitalized patients at 1 MPE were collected on average 28 ± 11 days after PCR-confirmation. Oropharyngeal (OP) and nasopharyngeal (NP) swab samples were collected at enrollment for all patients. Non-hospitalized patients were assigned World Health Organization (WHO) COVID-19 severity scores of 1–2, and moderate, severe, and deceased hospitalized COVID-19 patients were assigned WHO scores of 3–4, 5–7, and 8, respectively (**Supplementary Table 1**). For hospitalized patients, the severity scores used were maximum severity scores during their hospital stay. Samples were processed on the same day of collection and stored at −80°C until the time of the biological assays.

### Virus RNA levels

SARS-CoV-2 RT-PCR testing was performed on OP or NP swab samples using Abbott *m*2000 platform (Abbott Molecular, IL, USA) per the manufacturer instructions and as described previously^15,16^. SARS-CoV-2 viral RNA levels (copies/mL) were calculated from qPCR Ct values using the standard curve.

### SARS-CoV-2 variant inference

Likely variant of SARS-CoV-2 was inferred for each patient using the date of sample collection and the timeframe of variants during which community prevalence was above 95% according to Robinson, et al^17^. The ancestral variant was prevalent from January 18, 2021, to July 31, 2021.

### Cytokine/chemokine detection

Plasma proinflammatory cytokines and chemokines were measured using a custom multiplex kit from Meso Scale Discovery (MSD; Rockville, MD) according to the manufacturer’s instructions, and as described previously^14,18^. Cytokine and chemokine data were first shifted by a pseudo count of +1 to avoid zeros and then log_2_-transformed to have normal distributions. Analytes with signal below background were set to 0 and lower limits of detection were based on manufacturer’s recommendations.

### Binding antibody measurement by ELISA on plasma samples

Binding antibodies in plasma samples were determined using in-house ELISAs as described previously^8,19,20^. The 96-well plates (Immulon 4HBK, Thermo Fisher Scientific) were coated overnight at 4°C with 50μL of 2μg/mL of either Spike (S), spike receptor binding domain (S-RBD), or 1μg/mL of nucleocapsid (N) antigen diluted in 1X phosphate-buffered saline (PBS). Antigens were either engineered at Johns Hopkins University^8^ or were obtained through the National Cancer Institute Serological Sciences Networks (SeroNet) for COVID-19^21^. Plates were washed 3 times with 200μL of wash buffer (PBS with 0.1% Tween-20) and then blocked with 3% milk powder in PBS with 0.1%Tween-20 (PBS-T) for 1h at room temperature (RT). Heat-inactivated plasma samples were three-fold serially diluted 10 times, starting with 1:20 dilution in dilution buffer (1% milk + 0.1% PBS-T). The blocking buffer was removed and 100μL of diluted plasma samples were transferred. Plates were incubated for 2h at RT, washed, and 50μL of anti-human HRP IgG (1:5000, #A18823, Invitrogen, Thermo Fisher Scientific), IgA (1:5000, #A18787, Invitrogen, Thermo Fisher Scientific), IgG1 (1:4000, #9054–05, Southern Biotech), IgG2 (1:4000, #9060–05, Southern Biotech), IgG3 (1:4000, #9210–05, Southern Biotech) or IgG4 (1:8000, #9200–05, Southern Biotech) secondary antibody was added. After 1h incubation at RT, plates were washed, and 100μL of Sigmafast OPD (o-phenylenediamine dihydrochloride) solution (MilliporeSigma) was added. After 10 minutes of incubation at RT, the reaction was stopped by adding 50μL of 3M HCL (Thermo Fisher Scientific) and the plates were read for OD values at 490nm wavelength on a SpectraMax i3 ELISA plate reader (BioTek Instruments). Background-subtracted optical density values were plotted against the dilution factor to calculate the area under the curve (AUC). Spike and N IgG antibodies were converted into the international binding assay units (BAU/mL) using the standards calibrated at the Johns Hopkins University through the SeroNet assay harmonization project^21^. AUC and BAU/mL values were log-transformed for analysis. Limit of detection (LOD) was determined as half of the lowest BAU for the sample with a detectable titer (i.e., titer ≥20), while samples with undetectable titers (i.e., <20) received a value that was half the limit of detection^19^.

### ACE2 binding inhibition antibody assay

ACE2 binding inhibition antibody assay was performed using MSD V-PLEX SARS-CoV-2 ACE2 kits (Panel 29) according to the manufacturer’s protocol^19^. Antigen pre-coated plates were washed and incubated with plasma samples (1:100 dilution) for 1h followed by addition of SULFO-TAG conjugated human ACE2 protein for 1h at RT. After incubation, plates were washed, buffer was added, and plates were read with a MESO QuickPlex SQ 120 instrument. ACE2 binding inhibition activity corresponding to 1μg/mL of monoclonal antibody to the ancestral strain of SARS-CoV-2 S protein was determined using an 8-point calibration curve included in each plate. Percent inhibition was determined based on the equation ([1 – average sample electrochemiluminescence/average electrochemiluminescence signal of blank well] × 100) provided by the manufacturer.

### Complement activation assay

Complement activation assays were performed from plasma samples as described^22^ with modifications. Nunc MaxiSorp flat-bottom 96-well plates were coated with 100ng/well of S, S-RBD, or PBS alone. After overnight incubation, plates were washed with 0.1% PBS-T and blocked with 1% gelatin/PBS-T for 1h at RT. 100μL of heat-inactivated patient plasma diluted at 1:1000 in 1% gelatin/PBS-T were added to the wells and incubated for 1h at RT. After washing with PBS-T, normal human serum (NHS, Comptech) at 1:50 dilution in gelatin veronal buffer with calcium and magnesium (GVB^++^, Comptech) was added as the complement source. To remove any background anti-spike IgG response, total IgG was removed from NHS source. NHS was diluted 1:50 in GVB^++^ and incubated with increasing amounts of PureProteome protein A/G mix magnetic beads (Millipore) for 1h at 4°C with continuous mixing. Total IgG and anti-S background antibodies were fully removed, without affecting complement activity, using 50μL beads per 300μL of diluted NHS. After 1h incubation with NHS at 37°C, wells were washed with PBS-T, and goat anti-human C1q (Comptech, A200) diluted 1:20,000 in PBS-T was added for 1h at RT. HRP-labelled anti-goat IgG (Thermo Fisher Scientific, A16005) diluted 1:5000 in PBS-T was used as secondary antibody and incubated for 1 hour at RT. Following addition of SureBlue peroxidase reagent (IPL), reactions were stopped with HCL and absorbances were read at 450nm. Arbitrary units (AU) were calculated using a standard minus background binding to PBS-coated wells.

### Antibody-dependent cell-mediated cytotoxicity (ADCC) assays

Tet-on HEK-293 cells engineered to express Wuhan-1 S protein (hereafter HtetZ/SW1 HEK293 cells)^23^ in response to doxycycline (DOX) were incubated overnight with 1μg/mL DOX in DMEM containing 10% fetal bovine serum (FBS), 1% penicillin/streptomycin (P/S), zeocin (200μg/mL) and puromycin (3μg/mL). HtetZ/SW1 HEK-293 cells were detached with trypsin/EDTA, resuspended at 2 × 10^6^ cells/mL in Iscove’s Modified Dulbecco’s Medium (IMDM) (10% FBS and 1% P/S), and spike surface expression was confirmed by flow cytometry using commercial SARS-CoV-2 2019-nCoV spike S2 antibody (Sino Biological 40590-D001) and using purified IgG from anti-S positive patient plasma (n=3; **Supplementary Figure 2**). ADCC assays were performed in 96-well round bottom plates by incubating 50μL HtetZ/SW1 HEK-293 cells with 1μg of IgG purified from patient plasma (Melon Gel Spin Plate Kit, Thermo Fisher Scientific) After 30-minutes at 37°C, 50μL of Jurkat-Lucia^™^ NFAT-CD16 cells (InvivoGen) at 4 × 10^6^ cells/mL were added per well (effector: target ratio 2:1), mixed and centrifuged for 1-minute, at 800rpm. After 5-hour incubation at 37°C, 20μL of supernatant was collected and mixed with 50μL of QuantiLucTM solution (InvivoGen) in a 96-well black polystyrene plate (Corning Costar) to assess luciferase activity. A pool of high titer anti-S IgG purified from patient plasma was used to generate a standard curve to calculate the unknown sample AU and calibrate across plates.

### Multiplex antibody assays on mucosal samples

IgG and secretory IgA (sIgA) antibody responses on NP and OP swabs were determined using multiplex SARS-CoV-2 antibody assays as described^24–26^. The SARS-CoV-2 multiplex assay included two SARS-CoV-2 N antigens, two S, three S-RBD antigens, endemic coronavirus OC43, NL63, HKU1 and 229E antigens, respiratory syncytial virus (RSV), and several control beads (total IgG, IgA, IgM, BSA). Mucosal samples were added to assay buffer (PBS with 0.05% Tween 20 and 0.1% BSA) containing 1000 beads per bead set in each well of a 96-well plate. NP and OP swabs were tested at a 1:2 dilution for IgG and a 1:4 for sIgA. After a 1h sample incubation beads were washed twice, then phycoerythrin (PE)-labeled anti-human IgG or mouse anti-secretory component antibody, followed by PE-labeled anti-mouse antibody was added. After another 1-hour incubation beads were washed twice again and then read on a MagPix.

### Statistical analyses

All antibody (i.e., mucosal and serum antibodies measured as either AUC, BAU/mL, AU, or MFI) and virus RNA (copies/mL) data were log_10_-transformed. To account for possible zeros, complement and ADCC data (AU) were shifted by +1 prior to logarithmic transformation. ACE2 inhibition data (%) were arcsine transformed to be more consistent with the Gaussian assumptions used in analyses. The null hypotheses that hospitalization group means were equal at each timepoint were tested using Welch’s ANOVA with Benjamini-Hochberg post-hoc corrections at a 0.1 false discovery rate (FDR). Spearman correlation was used to quantify the association of viral load between nasal and oral samples and association of complement C1q with binding antibodies. Linear mixed-effects regression modelling was used to compare antibody trajectories over days from enrollment across COVID-19 disease severity groups. The results were visualized by plotting the estimated fixed effects against days since enrollment for different severity groups. The null hypothesis that all groups had the same dependence on time was tested using a likelihood ratio test comparing mixed effects models with and without the group by time interaction. Binding, complement, and ACE2 inhibition antibody data were scored by quartiles from 0–3 with data in the lower 25^th^ percentile scored as 0 and those in upper 75^th^ percentile scored as 3. Data were then totaled by antibody type (e.g., anti-N IgG, anti-S IgG, anti-S-RBD IgG, and anti-S-RBD IgA quartile scores were totaled by participant for an overall binding antibody score) to create an index score. Logistic regression models, with death as the binary outcome, were used against antibody scoring to evaluate how antibody levels were associated with the probability of death at enrollment or 1 MPE. A random forest algorithm was used to compare the predictive power of demographic (i.e., age, BMI, race/ethnicity, sex, and comorbidities) and serological variables for intubation or death as represented by the variable importance plots. Performance of random forest algorithms was assessed by receiver operating characteristic (ROC) curves and their AUC values for out of bag predictions (OOB) All p-values <0.05 were considered statistically significant. Statistical analyses were conducted in Stata 17.0, GraphPad Prism, and R.

## Results

### Demographic characteristics of the COVID-19 study cohorts

A total of 73 (46 female; 27 male) non-hospitalized and 105 (48 female; 57 male) hospitalized COVID-19 patients were included (**Table 1**). Out of the hospitalized patients, 41 (18 female, 23 male) were in the WHO moderate disease category, 40 patients (20 female; 20 male) were in the severe disease category, and 24 patients (10 female; 14 male) were deceased. For non-hospitalized patients, samples were collected for the ‘1 MPE’ timepoint at 46 ± 15 days and neither anti-N IgG nor anti-S IgG responses correlated with the number of days post-enrollment (**Supplementary Figure 1A-B**). Sample collection from hospitalized patients at the 1 MPE timepoint averaged at 28 ± 11 days (**Supplementary Figure 1C-D**). The number of days from hospital enrollment to death among the deceased cohort ranged between 3 to 261 days, with 75% of those dying from COVID-19 within 66 days of enrollment (**Supplementary Figure 1E**). For longitudinal analyses and predictive modeling, data from hospitalized patients that died within 100 DPE (n = 18) were included.

### Proinflammatory cytokine/chemokine, but not viral RNA, levels at enrollment are greater among hospitalized patients with more severe COVID-19

**Figure 1:**
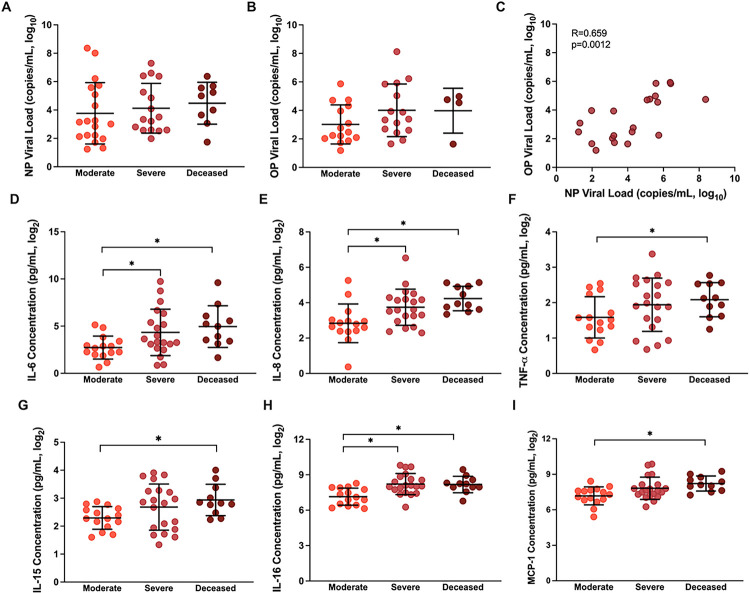
SARS-CoV-2 virus RNA and cytokine/chemokine responses among hospitalized COVID-19 patients at enrollment. (A) Nasopharyngeal (NP) viral load (copies/mL, log_10_) and (B) oropharyngeal (OP) viral load (copies/mL, log_10_) were measured by qPCR at enrollment and compared among patients classified as moderate (WHO score 3–4), severe (WHO score 5–7), or deceased (WHO score 8). (C) The Spearman correlation between OP and NP viral loads at enrollment. (D-I) Concentrations (pg/ml) of several proinflammatory cytokines and chemokines that differed among COVID-19 hospitalized patients classified as moderate, severe, or deceased. Data are presented as means with standard deviations in black. Asterisk (*) indicates statistically significant differences (p<0.05) by Welch’s ANOVA.

Virus RNA quantification was performed in OP and NP swabs collected from the hospitalized patients during enrollment. Viral RNA copy numbers did not differ among moderate, severe, and deceased patients in either NP or OP swab samples (p>0.05, Figure 1A–B. Virus RNA levels in OP and NP swabs were positively correlated (Spearman R=0.659, p=0.0023, Figure 1C). During enrollment, inflammatory cytokine/chemokine response levels in plasma were compared among hospitalized patients with different COVID-19 disease severities (**Supplementary Table 2**). Consistent with previous reports^27,28^, patients with severe disease (WHO score 5–7) or those who subsequently died from COVID-19 (WHO score 8) had greater concentrations of proinflammatory cytokines and chemokines, including IL-6, IL-8, TNF-a, IL-15, IL-16, and MCP-1, than hospitalized patients with moderate disease (WHO score 3–4) (Figure 1D–I, p<0.05 in each case).

### COVID-19 disease severity is not associated with mucosal antibody responses in hospitalized patients

**Figure 2:**
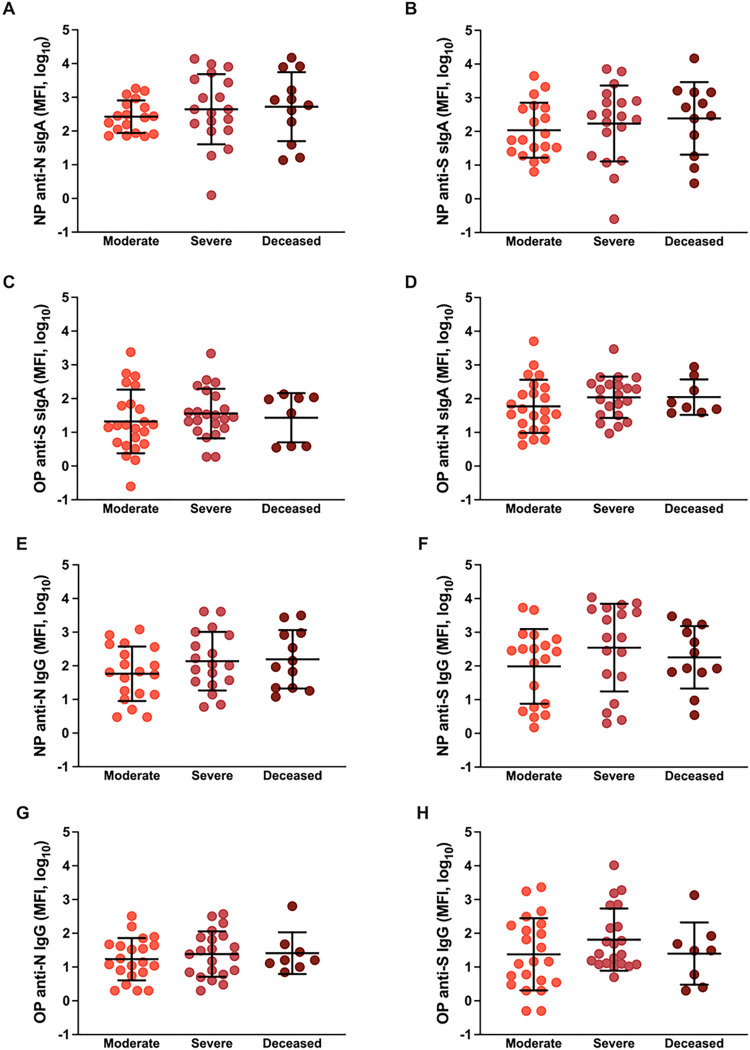
Mucosal antibody responses among hospitalized COVID-19 patients at enrollment. (A-D) Anti-nucleocapsid (N) and anti-Spike (S) secretory IgA or (E-H) IgG responses were measured as median florescence intensity (MFI) in nasopharyngeal (NP) or oropharyngeal (OP) samples and compared among COVID-19 hospitalized patients classified as moderate (WHO score 3–4), severe (WHO score 5–7), or deceased (WHO score 8). Means with standard deviations are depicted in all figures and data were analyzed using Welch’s ANOVA.

Using the OP and NP swab sample viral transport media collected during hospital enrollment, ancestral SARS-CoV-2 N- and S-specific IgG and secretory IgA (sIgA) antibody responses were measured. Binding antibody responses in mucosal samples against ancestral viral antigens did not differ based on COVID-19 disease severity among hospitalized patients (Figure 2A–H, p>0.05 in each case). The sIgA (**Supplementary Figure 3A-F**) and IgG (**Supplementary Figure 4A-F**) responses also were measured against other beta coronaviruses, including SARS, MERS, and HCoV, and OC43, in the NP and OP swab samples and were not significantly different among moderate, severe, and deceased patients in both OP and NP compartments. These data suggest mucosal antibody responses against SARS-CoV-2 do not differ by COVID-19 severity.

### Antibody responses are higher among hospitalized than non-hospitalized COVID-19 patients at 1 MPE

**Figure 3:**
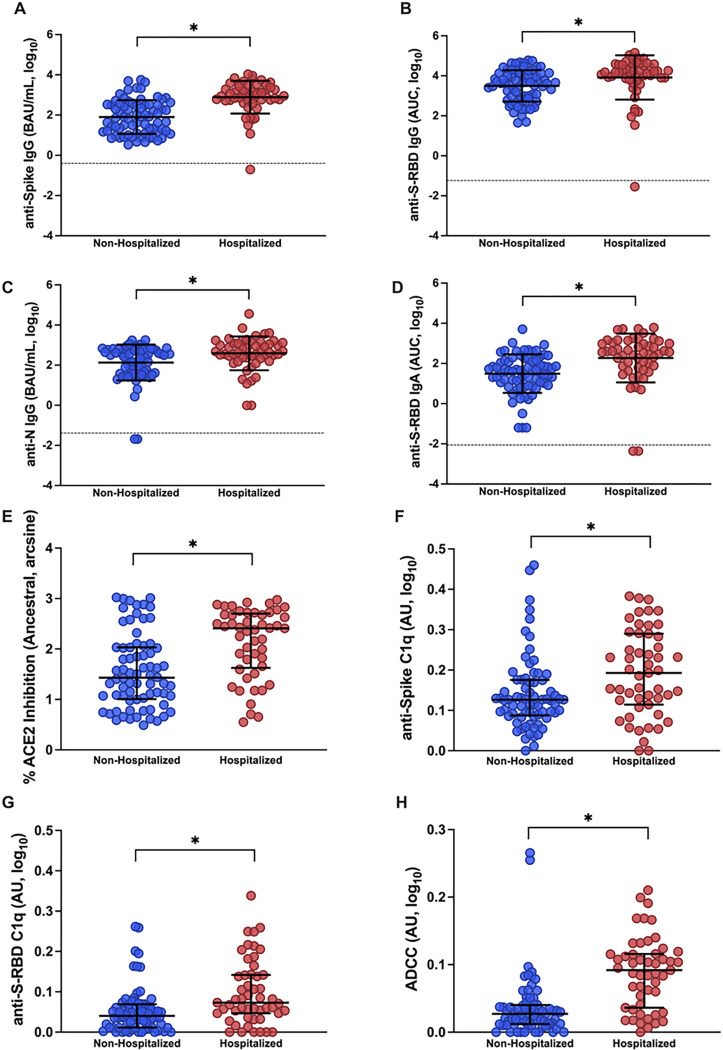
Antibody responses in plasma samples of non-hospitalized and hospitalized COVID-19 patients at 1-month post-enrollment (MPE). (A-D) IgG binding antibody responses against ancestral spike (S), spike receptor binding domain (S-RBD), and nucleocapsid (N) were quantified by ELISA and calculated as the binding antibody units (BAU) per ml if international standards were available or as the area under the curve (AUC) if standards were not available and titration curves only could be generated; (E) ACE2 binding inhibition antibodies were measured using MSD V-PLEX SARS-CoV-2 ACE2 kits; and (F-H) Fc effector antibody responses were quantified using complement fixation and antibody-dependent cellular cytotoxicity (ADCC) assays. All assays were run using ancestral SARS-CoV-2. Data were compared using Welch’s t-test to look at differences between unvaccinated non-hospitalized and hospitalized patients at 1 MPE. Means with standard deviations are depicted in black. Limit of detection (LOD) are indicated by the dashed lines. Comparisons were performed using Welch’s t-tests. Asterisk (*) indicates statistically significant differences (p<0.05).

Using plasma samples collected at 1 MPE, we compared antibody binding (i.e., anti-S IgG, anti-S-RBD IgG, anti-S-RBD IgA, and anti-N IgG), ACE2 binding inhibition, and Fc effector antibody responses (i.e., complement activation and ADCC) between non-hospitalized and hospitalized patients. Binding antibodies (Figure 3A–D) were significantly higher (p<0.05) among hospitalized patients than non-hospitalized patients at 1 MPE. Likewise, ACE2 binding inhibition antibody response were significantly higher (p<0.05) among hospitalized than non-hospitalized patients (Figure 3E). The Fc effector antibody functions, including complement activation as measured by anti-S and anti-S-RBD C1q antibodies (hereafter anti-S C1q and anti-S-RBD C1q, respectively), and ADCC, were significantly higher (p<0.05) in hospitalized than non-hospitalized patients (Figure 3F–H). Neither the reported sex (**Supplementary Figure 5**) nor age (**Supplementary Figure 6**) of the patients impacted binding, ACE2 binding inhibition, or Fc effector antibody responses among either non-hospitalized or hospitalized patients at 1 MPE in this cohort. Consistent with previous findings^8,9^, people who required hospitalization for acute COVID-19 had higher antibody responses at 1 MPE than patients who did not require hospitalization (Figure 3, **Supplementary Figures 5–6**).

### COVID-19 disease severity is correlated with plasma antibody responses over time until death or 100 DPE

**Figure 4:**
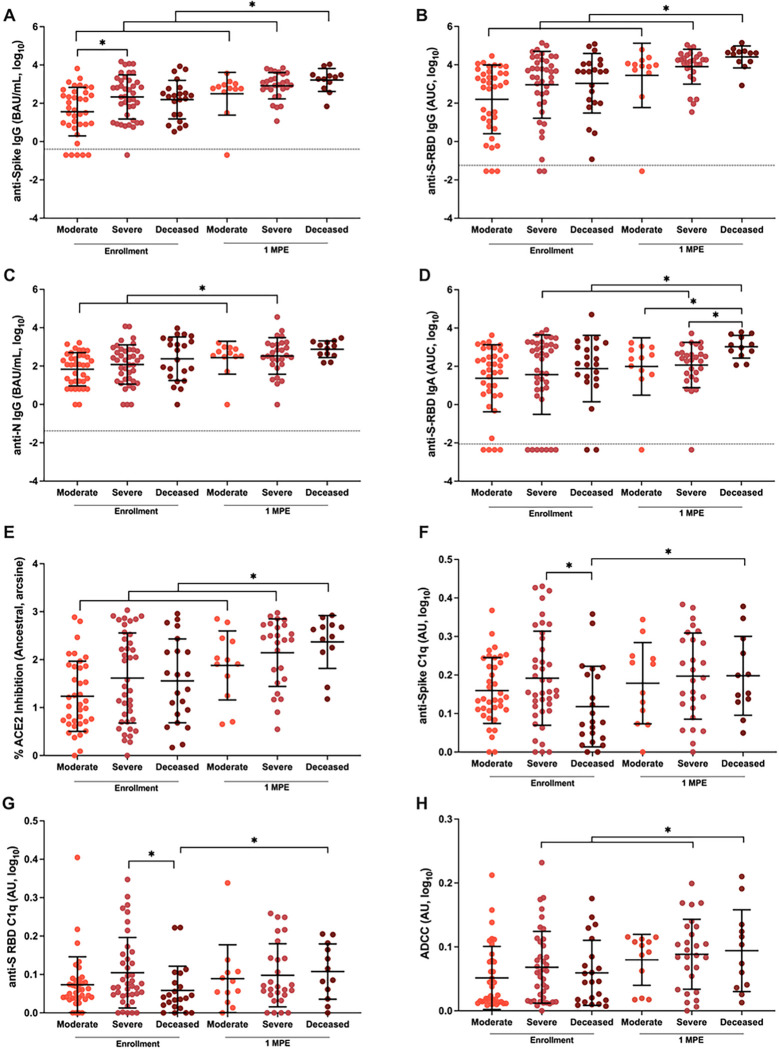
Binding, ACE2 inhibition, and Fc effector antibody responses in plasma among COVID-19 hospitalized patients at enrollment and 1-month post-enrollment (MPE). The binding (A-C) IgG and (D) IgA antibodies recognizing ancestral SARS-CoV-2 spike (S), spike receptor binding domain (S-RBD), or nucleocapsid (N) were quantified by ELISA, and measured as the binding antibody units (BAU) per ml if international standards were available or as the area under the curve (AUC) if standards were not available and titration curves could only be generated. (E) The percentage of ACE2 inhibition for the ancestral SARS-CoV-2 variant was calculated and arcsine transformed for analyses. (F-H) The Fc effector antibody responses were measured based on C1q complement fixation in response to either the spike or S-RBD or antibody dependent cellular cytotoxicity and reported as arbitrary units (AU). Antibody responses were compared among COVID-19 hospitalized patients classified as moderate (WHO score 3–4), severe (WHO score 5–7), or deceased (WHO score 8) using samples collected at enrollment vs. 1 MPE. Data are presented as means with standard deviations in black. Asterisk (*) indicates statistically significant differences (p<0.05) by linear mixed-effects regression to compare change over time or Welch’s ANOVA. Limit of detection (LOD) are indicated by the dashed lines.

Binding and ACE2 binding inhibition antibodies were measured in plasma samples from hospitalized patients, collected at hospital enrollment and through subsequent death or 100 DPE. During enrollment, anti-S IgG, but not anti-S-RBD IgG, anti-S-RBD IgA, anti-N IgG, or ACE2 binding inhibition antibody responses, were significantly higher among patients with severe compared to moderate disease (Figure 4A–E, p<0.05). After 1 MPE, anti-S IgG (Figure 4A), anti-S-RBD IgG (Figure 4B), and ACE2 binding inhibition (Figure 4E) antibody responses significantly increased over time (p<0.05 in each case) among all hospitalized patients, with the deceased patients consistently maintaining the highest antibody responses. Anti-N IgG responses increased at 1 MPE among both severe and moderate disease patients but did not change among deceased patients (Figure 4C). Among hospitalized patients with severe disease or dying from COVID-19, anti-S-RBD IgA (Figure 4D) increased over time since enrollment. At 1 MPE, patients who died from COVID-19 had significantly greater anti-S-RBD IgA response than patients with moderate or severe disease (p<0.05). Unlike ancestral SARS-CoV-2 (Figure 4E), ACE2 inhibition antibodies against SARS-CoV-2 variants were comparable among hospitalized patients with varying severities of disease (**Supplementary Figure 7**).

**Figure 5:**
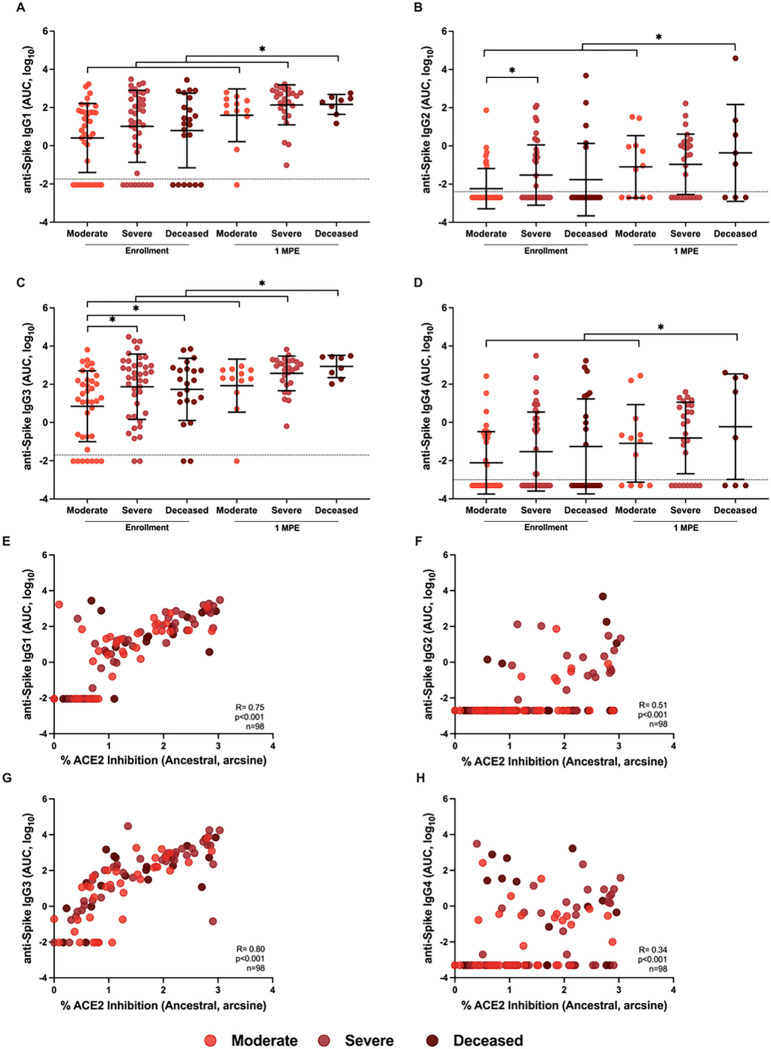
Analysis of anti-Spike (S) IgG subclasses (IgG1–4) among hospitalized COVID-19 patients at enrollment and 1-month post-enrollment (MPE). The binding of IgG1 (A), IgG2 (B), IgG3 (D), and IgG4 (D) to ancestral SARS-CoV-2 S antigen were measured as the area under the curve (AUC). Spearman correlation of IgG1 (E), IgG2 (F), IgG3 (G), and IgG4 (H) with % ACE2 inhibition at enrollment. Hospitalized COVID-19 patients were classified as moderate (WHO score 3–4), severe (WHO score 5–7), or deceased (WHO score 8). Data are presented as means with standard deviations in black. Limit of detection (LOD) are indicated by the dashed lines. Asterisk (*) indicates statistically significant differences (p<0.05) by linear mixed-effects regression to compare change over time or Welch’s ANOVA.

Because subclasses of IgG have different antibody effector functions, subclasses of IgG recognizing SARS-CoV-2 S were analyzed. At enrollment, anti-S IgG2 and IgG3 were significantly higher among either deceased or severe disease patients than moderate disease patients (Figure 5). From enrollment to 1 MPE, anti-S IgG1 and IgG3 levels significantly increased among all hospitalized patients, whereas anti-S IgG2 and IgG4 only increased over time among patients with moderate disease or those who died from COVID-19.

Because differential Fc effector antibody functions that mediate complement and innate immune cell activation can contribute to COVID-19 pathology^29–31^, anti-S C1q, anti-S-RBD C1q, and ADCC in plasma were measured among hospitalized patients at enrollment and 1 MPE (Figure 4F–H). At enrollment, anti-S C1q and anti-S-RBD C1q (Figure 4F–G) were significantly lower (p<0.05) in the patients who died from COVID-19 compared to hospitalized patients with severe disease. In contrast, ADCC responses were not significantly different among moderate, severe, and deceased patients (Figure 4H). Only deceased COVID-19 patients had a significant increase in anti-S C1q deposition (Figure 4F) and anti-S-RBD C1q deposition (Figure 4G) over time, from enrollment to 1 MPE (p<0.05). There was a significant increase in ADCC responses from enrollment to 1 MPE in patients with either severe disease or who died from COVID-19 (Figure 4H). The complement activity was primarily mediated by IgG rather than IgM antibodies as shown by the stronger correlation of complement with IgG than IgM (**Supplementary Figure 8A-H**). IgM antibodies, however, were better correlated with complement activity among hospitalized than non-hospitalized patients. Anti-S IgG1 and IgG3, but not anti-S IgG2 or IgG4, strongly correlated with anti-S C1q and anti-S-RBD C1q among hospitalized patients (**Supplementary Figure 8I-P**).

**Figure 6:**
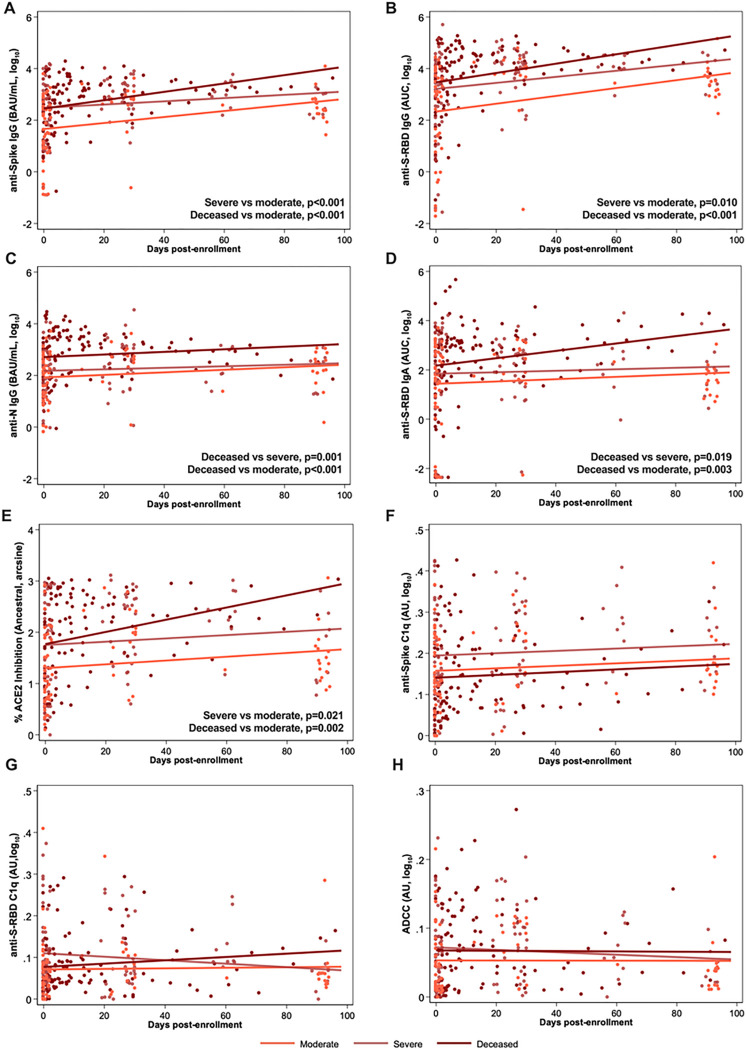
Antibody responses against ancestral SARS-CoV-2 over continuous days since enrollment until 100 days post-enrollment (DPE) or subsequent death among hospitalized COVID-19 patients. Linear mixed-effects regression models for (A-D) anti-spike (S), anti-spike receptor binding domain (S-RBD), or anti-nucleocapsid (N) IgG or IgA, measured as the binding antibody units (BAU) per ml if international standards were available or as the area under the curve (AUC) if standards were not available and only titration curves could be generated; (E) the percentage ACE2 inhibition against ancestral SARS-CoV-2 as a surrogate of virus neutralization, and (F-H) Fc effector antibody responses as measured by complement fixation against spike or S-RBD or antibody dependent cellular cytotoxicity (ADCC) up until 100 DPE or death among hospitalized patients classified as moderate (WHO score 3–4), severe (WHO score 5–7), or deceased (WHO score 8). Significant comparisons (p<0.05) by regression contrasts are shown within the figures.

With consideration of the antibody kinetics from days since enrollment until either death or 100 DPE among hospitalized COVID-19 patients, anti-S IgG, anti-S-RBD IgG, anti-N IgG, anti-S-RBD IgA, and ACE2 binding inhibition (Figure 6A–E) were maintained at higher levels over time among deceased patients as compared to other hospitalized patients. Fc effector activities, including anti-S C1q deposition, anti-S-RBD C1q deposition, and ADCC-mediating antibodies exhibited no changes over time among hospitalized patients (Figure 6F–H).

### Predictive value of plasma antibody titer as a biomarker for COVID-19-related death among hospitalized patients

**Figure 7:**
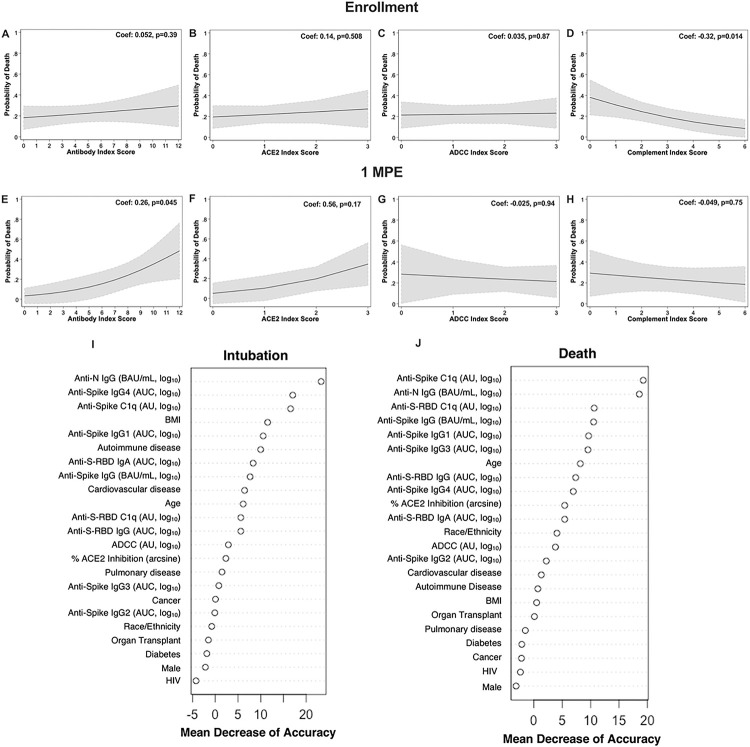
Logistic regression of COVID-19 death by indexed antibody variables among hospitalized COVID-19 patients at enrollment and 1-month post-enrollment (MPE). Logistic regression modelling for death among hospitalized COVID-19 patients by indexed scores based on quartiles of (A, E) binding, (B, F) ACE2 inhibition, (C, G) ADCC, or (D, H) complement fixation at enrollment or 1 MPE, respectively. Predicted probabilities from logistic regression models are graphed in black with 95% confidence intervals shaded in grey. (I-J) Random Forest variable importance plots were used to determine the relative ranking of different demographic and serological variables in descending order of importance, as expressed in mean percentage decrease accuracy, for model predictions of intubation or death among hospitalized patients at enrollment. Exclusion of variables of high mean decrease accuracy, particularly those >10%, would result in models that would less accurately classify patients as intubated or deceased.

We sought to understand the predictive value of antibody titers as a biomarker for subsequent death from COVID-19 among hospitalized patients (Figure 7A–H). A cumulative antibody score was calculated by first dividing each antibody measure into quartiles with assigned scores of 0 to 3, ranging from the lowest quartile to the highest quartile, and totaled across the measures by type of response (e.g., binding antibody index score is the sum of the quartile scores across anti-N IgG, anti-S IgG, anti-S-RBD IgG, and anti-S-RBD IgA). Using logistic regression modelling with death as a binary outcome against antibody scoring, greater cumulative binding antibody scores at 1 MPE were associated with an increased probability of death due to COVID-19 (Figure 7E), which was not observed at enrollment (Figure 7A). Similarly, a positive, but not statistically significant, association between the probability of death and ACE2 binding inhibition antibody scoring was observed at 1 MPE (Figure 7F), but not at enrollment (Figure 7B). The ability of anti-S antibodies to induce ADCC at either enrollment (Figure 7C) or at 1 MPE (Figure 7G) did not associate with death from COVID-19. Antibody-induced complement activation during enrollment (Figure 7D), but not at 1 MPE (Figure 7H), was negatively associated with probability of death due to COVID-19. Logistic regression models cannot establish a causative relationship between binding antibody levels or complement with subsequent death outcomes among hospitalized patients, but rather demonstrate an association that should be further investigated.

Random forest models were used to evaluate demographic (e.g., age, sex, BMI, race/ethnicity), clinical (e.g., diabetes, HIV, solid organ transplant, and other comorbidities.), and serological measures at enrollment as predictors of intubation or death among hospitalized patients. Using all complete data, the intubation model, comparing hospitalized patients who were intubated or not, had an ROC curve AUC value of 0.73 and, similarly, the model for death had an ROC curve AUC value of 0.71. For both intubation and death models, anti-N IgG antibodies and anti-S antibody-mediated complement fixation (anti-S C1q) were consistently prioritized as top variables that predicted intubation or death with the greatest mean decrease accuracy according to variance importance plots (Figure 7I–J). For the intubation model, anti-N IgG titers ranked first, anti-S IgG4 titers ranked second, anti-S C1q deposition ranked third, and BMI ranked fourth for predictive ability and were the top variables necessary for accurately classifying patients as intubated in our model (Figure 7I). For death from COVID-19, anti-S C1q deposition ranked first, anti-N IgG titer ranked second, anti-S-RBD C1q deposition ranked third, and anti-S IgG titer ranked fourth for predictive ability (Figure 7J). To further confirm these findings, we ran random forest models with either only demographic variables or serological variables. The ROC curve AUC value for the random forest intubation model with only demographic variables (0.54) was much lower than the random forest intubation model with only serological variables (0.69), indicating that performance of random forest models with only demographic variables is inferior to those with serological measures in our cohort. Overall, our models suggest that serological variables, particularly anti-N-IgG titer, and anti-S C1q deposition, were better able to classify the data for intubation or subsequent death compared to demographic and clinical variables at enrollment.

## Discussion

In the current study, patients who became severely ill or died from COVID-19 consistently maintained greater antibody responses compared to hospitalized patients with moderate disease or non-hospitalized patients. We utilized samples collected from peripheral blood and mucosal sites to analyze over 20 different antibody characteristics, including diverse antibody isotypes, virus neutralizing responses, and non-neutralizing activities, against multiple SARS-CoV-2 epitopes to provide a deep interrogation of the antibody landscape in a cohort of COVID-19 patients. Using machine learning and artificial intelligence (AI) algorithms, we identified the characteristics of the antibody landscape that could predict whether a patient would succumb to or recover from COVID-19.

Systemic complement activation and the ability of anti-S antibodies to induce ADCC were determinants of COVID-19 severity^29–32^. In our cohort, anti-S and anti-S-RBD antibody-mediated complement deposition was lower in hospitalized patients that died compared to hospitalized patients who recovered from COVID-19. We expected that Fc-mediated antibody functions would increase like anti-S and anti-S-RBD antibody titers among patients hospitalized with more severe COVID-19. In contrast, among patients with progressively worsening disease, antibodies to SARS-CoV-2 had a reduced capacity to activate complement and ADCC, which could contribute to a reduced ability to clear the virus. These findings highlight the need to better understand non-neutralizing functions of antibodies to SARS-CoV-2 during COVID-19, their predictive value for disease outcomes, and the mechanisms of functional heterogeneity.

Other studies have highlighted the importance of antibody biomarkers in defining the COVID-19 outcome although the results are inconsistent, likely due to differences in study design, patients’ characteristics (e.g., age, sex, ethnicity, etc.), antibody assays, and analytical methods (e.g., the groups with which comparisons are made). For example, Kritikos et al. showed an association of IgG antibody titer at the time of hospital admission with the requirement of mechanical ventilation, while Salgado et al. showed that antibody responses are not significantly different between discharged and deceased COVID-19 patients, except for antibodies towards disordered linker region of N protein^33,34^. De Vito et al. used multivariate cox regression modeling to show that anti-N IgG titers at hospital admission are independently associated with the risk of death from COVID-19^35^. Smit et al. showed a lower virus neutralizing antibody titer during hospital admission in fatal versus non-fatal cases of COVID-19^36^, while Garcia-Beltran et al. showed that reduced neutralization potency, but not neutralizing antibody titers, is associated with death from COVID-19^12^. Our data support and expand on previous studies by illustrating that elevated binding and virus neutralization and lower levels of complement fixing antibody, together with elevated cytokine and chemokine responses during enrollment, are associated with the likelihood of death and intubation from COVID-19 among hospitalized patients.

In our study, the novel application of machine learning algorithms, such as random forest models, allowed for the identification of the variables, including sociodemographic and immunological measures, that were most predictive of severe COVID-19 outcomes (i.e., intubation or not; deceased or not) in our dataset. Machine learning has been applied to -omic datasets and infectious disease studies with sociodemographic and clinical variables (e.g., age, sex, comorbidities, medications, vital signs, symptoms, lab tests, etc.); however, machine learning has been underutilized with immunological datasets^37–42^. In our study, upper respiratory tract viral RNA levels at the time of enrollment and sociodemographic factors, such as age, sex, or BMI, were not strongly predictive of intubation or death from COVID-19 relative to the serological variables in this dataset. Our machine learning models revealed that the strongest predictors of either intubation or death from COVID-19 among hospitalized patients were elevated IgG binding antibodies that recognize SARS-CoV-2 N and S proteins and virus-specific antibodies that activate complement deposition.

This study has limitations. We used samples of convenience collected during the pandemic. We did not perform sample size calculations; thus, our statistical modeling approaches and interpretations from this study may be influenced by the size of the datasets and the specific variables included. Additionally, hospitalized and non-hospitalized patients were enrolled through two separate parent studies and, therefore, sample collection was not designed to have these patient cohorts aligned for comparisons across all timepoints between these groups. Collection of demographic and clinical data (e.g., comorbidities) were conducted with separate surveys in these two parent studies and may differ in how comorbidities were defined.

Immunological datasets are often highly complex with diverse dependent measures across many sample types. The standard practice among researchers has been to perform over-simplified analyses, such as parametric or non-parametric pairwise comparisons and regression analyses, that may either limit the ability to identify critical associations or over-interpret them. Machine learning and AI methods now offer unique perspectives for interrogating data with a systems-level approach. Machine learning and AI can inform diagnoses, outcomes, therapeutic targets, and immune profiles for a wide range of diseases with significant applications in biomedicine and immunological research. We have taken a novel approach to show the application of machine learning and AI for finding serological biomarkers that predict outcomes during the COVID-19 pandemic, which has application for future pandemic preparedness.

## Data Availability

All data are available through the NIH/NCI Serological Sciences Network for COVID-19 (SeroNet) data repository, accessible via ImmPort Shared Data (ImmPort Seronet Search).
